# Autoimmune Hepatitis Leading to Liver Cirrhosis: A Case Report

**DOI:** 10.31729/jnma.7808

**Published:** 2022-12-31

**Authors:** Moon Shrestha, Sudip Chandra Subedi, Sangam Shah, Jeny Acharya, Milan Regmi, Neha Mehta

**Affiliations:** 1 Tribhuvan University Teaching Hospital, Maharajgunj, Kathmandu, Nepal; 2 Kanti Childrens Hospital, Maharajgunj, Kathmandu, Nepal; 3 Maharajgunj Medical Campus, Institute of Medicine, Maharajgunj, Kathmandu, Nepal

**Keywords:** *autoimmune hepatitis*, *case reports*, *chronic hepatitis*, *liver cirrhosis*

## Abstract

Autoimmune hepatitis is a rare form of chronic liver inflammation that begins as acute hepatitis and progresses to chronic liver disease. It presents with varied clinical features from acute hepatitis to chronic liver diseases like chronic viral hepatitis and alcoholic liver disease, making it difficult to diagnose in the absence of a high index of suspicion and adequate laboratory support. Autoimmune hepatitis is divided into two categories autoimmune hepatitis-1 and autoimmune hepatitis-2 based on the antibodies involved. We discuss the case of a 37-year-old woman who developed autoimmune hepatitis-1, with swelling and epigastric pain. These symptoms later progressed to liver cirrhosis leading to the death of the patient. Autoimmune hepatitis is extremely sensitive to immunosuppressive medication, it is necessary to maintain a high suspicion index for the disease because a prompt diagnosis can be an integral step toward a better prognosis of the disease.

## INTRODUCTION

Autoimmune hepatitis is a rare autoimmune disease that causes chronic liver damage and eventually leads to liver cirrhosis if not treated.^1^ Serological studies must rule out chronic hepatitis B and C, which are prevalent causes of cirrhosis in Asian nations, as well as nonalcoholic fatty liver disease in non-alcoholic patients.^2^ The clinical presentation varies from asymptomatic patients to acute hepatitis or chronic liver failure. Liver biopsy is important not only for diagnosis but also for determining the patient's prognosis and treatment options.^3^ Here, we report a case of a 37-year-old female with autoimmune hepatitis (AIH) who later developed liver cirrhosis resulting in the demise of the patient.

## CASE REPORT

A 37-year old female presented to our centre with chief complaints of generalised swelling of the body for 2 months and epigastric pain for 10 days. The swelling first appeared in the abdomen and later progressed to the entire body. It was non-painful and aggravated while standing for a long duration. Epigastric pain was non-radiating, was not associated with the intake of meals, and had no postural variation. She had a history of jaundice 2 months back. She had no fever, vomiting, hematemesis, melena, weight loss, and loss in appetite. Her bowel and bladder habits were normal. She had no history of blood transfusion, drug abuse, tattooing on the skin, sexual promiscuity or recent travel. She had diabetes mellitus type II for 1 year for which she was under insulin therapy. She did not smoke or consume alcohol.

During the initial visit, she was well oriented to time, place, and person, Glasgow Coma Scale (GCS) was 14/15. On general physical examination, icterus and bilateral pitting oedema were present, but there was no cyanosis, clubbing, or pallor. Her axillary temperature was 36.1^o^C blood pressure was 120/84 mm of Hg, heart rate was 86 beats per minute, and respiratory rate was 22 breaths per minute. Abdominal examination showed distension. Hepatomegaly and splenomegaly were noted on palpation. Other systemic examinations revealed normal findings.

Laboratory examination revealed haemoglobin 9.4 gm%, total leukocyte count was 4620 cells/mm^3^, neutrophil 72%, lymphocyte 21%, and platelet count 157000 cells/mm^3^. Her prothrombin time was 22 seconds and the international normalised ratio (INR) was 1.58. The alanine transferase (ALT) was 310 U/L, Aspartate Aminotransferase (AST) was 936 U/l, albumin level was 2.4 g/dl in serum, total and direct bilirubin level was 16.10 and 5.20 mg/dl respectively, and total protein was 6 g/dl. Random blood sugar level was normal (111 mg/dl) and serum levels of sodium, potassium, creatinine, and urea were normal. c-reactive protein (CRP) was 11 mg/L. Later, Immunoglobulin (IgG) level in the serum was 1095 mg/dl (700-1600 mg/dl). Anti hepatitis A virus IgM, and anti-hepatitis E virus IgM was negative while HbsAg and antinuclear antibody (ANA) were non-reactive. Similarly, serum antigens anti-Liver/Kidney microsomal (LKM), anti-mitochondrial antibody (AMA) were negative.

Serum markers anti-neutrophil cytoplasmic antibodies (c-ANCA), perinuclear anti-neutrophil cytoplasmic antibodies (p-ANCA), and anti-glomerular basement membrane (GBM) were negative while anti-smooth muscle antibody (ASMA) was positive.

**Figure 1:**
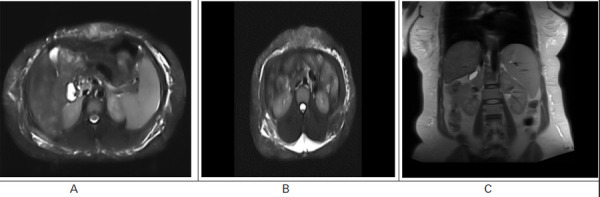
A) MRCP shows few collaterals in peripancreatic region, B) Left lumbar region draining into superior mesenteric veins, C) Splenomegaly.

Ultrasonography (USG) of the abdomen and pelvis showed absent flow in the right branch of the portal vein with few dilated collateral veins in the peripancreatic region, the gallbladder wall was thickened with sludge, and the uterus was bulky (9 x 5.6 x 5 cm) approximately. Magnetic resonance cholangiopancreatography (MRCP) revealed few collaterals in the peripancreatic region and left lumbar region draining into superior mesenteric veins, and splenomegaly (Figure 1).

**Figure 2:**
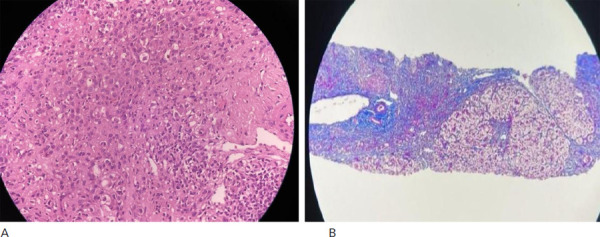
A) Marked interface hepatitis and lobular inflammation, composed predominantly of lymphocytes, plasma cells and few neutrophils, B) The biopsy of the liver showed multiple cores of liver parenchyma with prominent bridging fibrosis with nodule formation highlighted by Masson's trichrome.

The biopsy of the liver showed multiple cores of liver parenchyma with prominent bridging fibrosis with nodule formation highlighted by Masson's trichrome. There was marked interface hepatitis and lobular inflammation that was composed predominantly of lymphocytes, plasma cells and few neutrophils. The hepatocytes show marked feathery degeneration with rosette formation (Figure 2).

Based on the biopsy findings she was diagnosed with autoimmune hepatitis type I. She was given IV (Intravenous) ceftriaxone and prednisolone for initial 4 days. She then suddenly had decreased blood pressure and urine output, altered sensorium suggestive of shock. She was transferred to the intensive care unit where IV meropenem was given. She was kept on nil per oral (NPO) with a nasogastric tube. She suddenly became decompensated and disoriented. She developed bilateral pneumonia, had low blood pressure, and had low urine output. Even with inotropes support her blood pressure was low. She went into septic shock and finally, she died after 15 days of hospital stay.

## DISCUSSION

The precise pathophysiologic mechanism of AIH is unknown. The most widely accepted theory is that AIH is a disease that develops in a genetically susceptible individual who is also exposed to environmental triggers.^4^ As a result, the autoimmune onslaught is maintained, potentially through molecular mimicry, and is aided by the loss of regulatory T-cell control. Drugs, viral antigens (hepatitis A, B, and C), and herbal compounds have all been found as triggers. The immunologic tests serum ANA, smooth muscle antibody (SMA), anti-LKM1, and IgG are required for the diagnosis of AIH. Anti-actin antibodies, anti-LKM3, anti-liver cytosol 1, pANCA, and antibodies to soluble liver antigen (SLA) or liver pancreas antigen are all immunological tests that may be relevant (LP).^4-6^

The mild rise of transaminases, lower limit serum albumin, and high total protein levels in this patient indicated persistent hepatocyte inflammation (indicating hypergammaglobulinemia). Despite the patient's negative ANA and serum IgG, a positive SMA was enough to diagnose AIH, specifically type 1, in a patient who had no history of alcohol consumption and no hepatitis B or C serological markers. Despite the side effects, the initial spectacular response to prednisolone medication was highly encouraging in a case report. After 1 year of immunosuppressive medication, albumin levels improved dramatically, the globulin component of total protein, ALT, AST, and INR were normalised, and bilirubin levels were nearing normal.^7^ However, our patient did not respond to prednisolone from the initial stage.

AIH can exhibit cholestatic symptoms that are similar to primary sclerosing cholangitis or primary biliary cirrhosis, and overlap with these diseases has been reported in 10-20% and 2-8% of cases, respectively.^6^ The existence of conjugated hyperbilirubinemia and the finding of a biliary tree on abdominal ultrasonography, however, ruled out sclerosing cholangitis and primary biliary cirrhosis. Past history of jaundice, pedal oedema, ascites, hepatomegaly, splenomegaly, and coarsened liver on ultrasonography all indicate that this patient's condition was chronic and that she had developed liver cirrhosis.

Anti-inflammatory or immunosuppressive medications have traditionally been used to treat both type 1 and type 2 diseases. When properly treated, all treated patients have a 20-year survival rate of more than 80%, and their life expectancy is comparable to that of age and sex-matched normal subjects from the same geographic location. The treatment is separated into two stages: (i) induction of remission and (ii) maintenance of remission.^5,6^ Alternative induction therapies for AIH include prednisolone monotherapy and prednisolone in combination with azathioprine (AZA); for maintenance, Prednisolone in combination with AZA and AZA monotherapy is preferable to prednisolone monotherapy. Prednisolone-related adverse effects are reduced more effectively with combination therapy. Budesonide can be used instead of prednisolone to decrease steroid-related adverse effects.^4,6^

Remission in previously symptomatic individuals is defined as a complete normalisation of all inflammatory parameters, such as AST, ALT, bilirubin, and IgG, as well as symptom relief and normal liver histology, for at least 2 to 3 years.^8^ After stopping treatment, some patients may relapse. The use of a prednisolone/azathioprine combination therapy after the first episode of recurrence is ideal.^5^ Tacrolimus, mycophenolate mofetil, cyclosporine, methotrexate, cyclophosphamide, ursodeoxycholic acid, infliximab, and rituximab are among the alternative therapy for relapse. Although each of the drugs has shown some promising results, the therapies have not been standardised due to a lack of data from randomized controlled studies.^5,9^

Although liver transplantation is the most effective treatment for all types of liver illness, it has a modest role in AIH. AIH is responsible for about 4% of liver transplants in the United States and Europe. The avoidance of liver transplantation through earlier diagnosis and sufficient immunosuppressive medication is, in fact, the cornerstone of AIH management. Patients who are resistant to or intolerant to immunosuppressive medication, as well as those who develop end-stage liver disease, require liver transplantation.^8^

Since AIH is extremely sensitive to immunosuppressive medication, it is necessary to maintain a high suspicion index for the disease because a prompt diagnosis can be an integral step toward a better prognosis of the disease. We need to properly assess all chronic liver disease cases of apparently unknown aetiology for AIH in order to prevent progression to end-stage liver disease.
